# Webinar fatigue: fallout of COVID-19

**DOI:** 10.1186/s42506-021-00069-y

**Published:** 2021-04-16

**Authors:** Manoj Kumar Sharma, Shweta Sunil, Nitin Anand, Senthil Amudhan, Sundarnag Ganjekar

**Affiliations:** 1 Department of Clinical Psychology, SHUT clinic (Service for Healthy Use of Technology), NIMHANS Centre for Well Being, National Institute of Mental Health & Neuro Sciences, Bengaluru, Karnataka India; 2 Department of Psychology, CMR University, CMR Group of Institutions, Bengaluru, Karnataka India; 3 Department of Clinical Psychology, National Institute of Mental Health & Neuro Sciences, Bengaluru, Karnataka India; 4 Department of Epidemiology, National Institute of Mental Health & Neuro Sciences, Bengaluru, Karnataka India; 5 Department of Psychiatry, National Institute of Mental Health & Neuro Sciences, Bengaluru, Karnataka 560029 India

Dear Editor,

Webinar fatigue is a new phenomenon that has emerged due to the COVID-19 lockdown where there is an increased use of online/web meetings or other modalities for work, academics, and learning new skills in leisure time. This change has led to increased environmental demands associated with near-constant elevated levels of physiological activation, heightened attention, and increased pressure to respond and perform remotely with higher efficiency, subjective sense of restlessness, anxiety, and inability to relax. Participation in webinars involves maintaining eye-contact, sustained attention, maintaining an upright body posture, controlling one’s facial expressions to appear consistently engaged for extended periods of time. The webinars and web-based meetings require greater attention towards the verbal and non-verbal responses from all participants in comparison to in-person meetings. At times, the minimal technical delays in receiving responses from others are negatively perceived. It makes the receiver perceive the individual on the other end as less focused, less courteous, and less friendly. These changes in work requirements during the COVID-19 has led to the physical distress/digital fatigue in the form of eye strain, pain in the neck, back and shoulders, increased sensitivity to light, and decreased and disturbed sleep. Webinar fatigue is now becoming fairly common among 75% of people who use two or more gadgets simultaneously, and among 53% of people who use one gadget at a time [1].

Besides, it is often difficult to create a distraction-free space in one’s home for extended periods of 8 to 10 h and sometimes for the whole day. Attending webinars from home also requires an increased self-control to stay focused and not get distracted by events within the home setting. These accommodations and continuous participation in the webinars from home often add to the sense of dissatisfaction with the information, distress resulting from loss of time in an unproductive task, screen-fatigue, physical exhaustion, mental exhaustion, and ultimately digital burnout [1, 2]. The continual experience of distressing symptoms further leads to dissatisfaction with work, irritability, lowered productivity, fatigue, negativity, cynicism, and ultimately burnout [3, 4]. The pathway to digital fatigue and burnout appears to be accelerated by excessive use of digital devices for webinars in the times of COVID-19 lockdown. The 11th revision of the International Classification of Diseases (ICD-11) includes burnout as an occupational phenomenon rather than a medical condition [5]. This highlights the crucial role of workplaces in mitigating webinar fatigue.

## Conclusions

In the current scenario, wherein the evidence-based treatment for COVID-19 appears at least many months away, a systematic and multifaceted approach is required to mitigate the webinar fatigue and burnout. Webinar hosts should be trained in adopting best practices, promoting group interactions and breaking longer content while the end-users should be educated to embrace digital hygiene as a modality to overcome the risk of webinar fatigue and burnout. Awareness about webinar fatigue and the importance of “less is more” and “quality rather than frequency” with regard to webinars should be campaigned massively. There is a need for webinar fatigue risk management system that includes education, policies, practices, and environmental changes for prevention, early diagnosis, and management.

## Data Availability

Not applicable

## Abbreviations

ICD-11: International Classification of Diseases-11
COVID-19: Coronavirus disease
SARS-CoV-2: Severe acute respiratory syndrome coronavirus 2
